# Design of Fe-Co-Cr-Ni-Mn-Al-Ti Multi-Principal Element Alloys Based on Machine Learning

**DOI:** 10.3390/ma19020422

**Published:** 2026-01-21

**Authors:** Xiaotian Xu, Zhongping He, Kaiyuan Zheng, Lun Che, Feng Zhao, Deng Hua

**Affiliations:** 1 School of Mechanical Engineering, Chengdu University, Chengdu 610106, China; 2 Institute for Advanced Study, Chengdu University, Chengdu 610106, China; 3 School of Artificial Intelligence and Electronic Engineering, Sichuan University of Business and Technology, Chengdu 611745, China

**Keywords:** multi-principal element, machine learning, prediction, design, mechanical properties

## Abstract

Machine learning has been widely applied to phase prediction and property evaluation in multi-principal element alloys. In this work, a data-driven machine learning framework is proposed to predict the ultimate tensile strength (UTS) and total elongation (TE) of Fe-Co-Cr-Ni-Mn-Al-Ti multi-principal element alloys (MPEAs), offering a cost-effective route for the design of new MPEAs. A dataset was compiled through an extensive literature survey, and six different machine learning models were benchmarked, from which XGBoost was ultimately selected as the optimal model. The feature set was constructed on the basis of theoretical considerations and experimental data reported in the literature, and SHAP analysis was employed to further elucidate the relative importance of individual features. By imposing constraints on the screened features, two alloys predicted to exhibit superior performance under different heat-treatment conditions were identified and fabricated for experimental validation. The experimental results confirmed the reliability of the model in predicting fracture strength, and the errors observed in ductility prediction were critically examined and discussed. Moreover, the strengthening mechanisms of the designed MPEAs were further explored in terms of microstructural characteristics and lattice distortion effects. The alloy design methodology developed in this study not only provides a theoretical basis for exploring unexplored compositional spaces and processing conditions in multi-principal element alloys, but also offers an effective tool for developing novel alloys that simultaneously achieve high strength and good ductility.

## 1. Introduction

The pursuit of high-performance materials constitutes a central objective in alloy design. Among the various mechanical properties, strength and ductility are paramount, as they directly dictate a material’s suitability for specific applications. However, overcoming the inherent trade-off between these two properties remains a classic challenge. Conventional alloys, typically based on a single principal element with minor additions, derive their properties predominantly from that base element. In contrast, multi-principal element alloys (MPEAs) [1,2,3,4,5,6,7,8,9,10] represent a novel material class, generally composed of five or more elements in near-equiatomic proportions, where properties emerge from synergistic multi-element interactions. Benefiting from the so-called “high-entropy effect,” MPEAs can circumvent limitations imposed by the traditional Gibbs phase rule, often stabilizing simple solid-solution phases. Coupled with pronounced lattice-distortion effects, these characteristics can yield superior mechanical properties [11,12,13,14,15,16], creating new avenues for materials design.

A milestone was reached in 2004 with the proposal of the equiatomic Fe-Co-Cr-Ni-Mn “Cantor” alloy [1,2], which forms a single face-centered cubic (FCC) phase and exhibits exceptional strength, ductility, and fracture toughness at cryogenic temperatures. However, its outstanding performance is largely confined to low-temperature regimes, limiting room-temperature applicability. Subsequent research has therefore focused on developing equiatomic solid-solution variants and non-equiatomic derivatives of the Cantor system to enhance room-temperature mechanical properties. Studies by Wu et al. on the temperature dependence of FCC equiatomic solid-solution alloys revealed that while the strength of Ni-Co and Ni-containing alloys is relatively temperature-insensitive, Cr-containing alloys generally exhibit higher strength, suggesting potential advantages for non-equiatomic compositions [17]. For instance, Li et al. successfully designed a metastable dual-phase Fe_50_Mn_30_Co_10_Cr_10_ high-entropy alloy that effectively balances strength and ductility [18]. Concurrently, advances in processing technologies have enabled conventional alloys to achieve improved strength–ductility combinations at competitive costs. Consequently, further enhancing the room-temperature performance of MPEAs has become increasingly urgent.

To address this, design strategies from conventional alloys, such as deliberate alloying additions, are being applied to MPEAs. Al and Ti are commonly introduced into Cantor-derived alloys to enhance strength through pronounced lattice distortion (solid-solution strengthening) and the formation of ordered precipitates (e.g., L1_2_ or B2-type) under appropriate heat treatments. These nanoscale ordered domains can impede dislocation motion effectively while a ductile FCC matrix is retained, offering a feasible route toward improved room-temperature strength. Several systems, including Fe-Co-Cr-Ni-Mn-Al-Ti [19], Fe-Co-Cr-Ni-Al [20], Co-Cr-Ni-Ti [21], and Co-Cr-Fe-Ni [22], have been developed, expanding the compositional landscape and exploring performance potential. Nonetheless, the vast compositional space of MPEAs, encompassing tens of thousands of possible elemental combinations, renders traditional empirical trial-and-error approaches increasingly inefficient, particularly regarding development time and cost. There is a pressing need for efficient design strategies that minimize experimental iterations and accelerate the discovery of new MPEAs. The rapid advancement of machine learning (ML) offers a powerful solution. Fueled by progress in computational tools, alloy design is transitioning from trial-and-error exploration towards demand-driven, property-oriented design—a key driver of modern industrial innovation. By constructing high-performance computational models trained on existing data, ML enables accurate prediction of thermal stability, phase formation, and mechanical properties for diverse MPEA compositions, facilitating systematic exploration of their vast compositional space [7,23,24,25,26,27,28,29,30,31,32,33].

Significant progress has been made in applying ML to explore MPEA compositions. For example, Wang et al. employed a neural-network model combined with conditional random search for inverse design within a broad compositional space, identifying two high-entropy alloys that were experimentally validated to exhibit excellent strength and ductility [34]. Singh et al. integrated an evolutionary data-driven model with bi-objective genetic programming and a genetic algorithm to predict and optimize the yield strength and ductility of Al-Cr-Fe-Co-Ni-Cu alloys, achieving a designed alloy with a strength of 1795 ± 21 MPa and 31.45% ductility [35]. However, ML studies specifically targeting the Fe-Co-Cr-Ni-Mn-Al-Ti MPEA system remain relatively scarce. This work therefore focuses on this system for ML-based prediction and experimental validation.

In this study, 18 features encompassing alloy composition, thermodynamic parameters, and processing conditions were selected to characterize the key factors governing the mechanical properties of Fe-Co-Cr-Ni-Mn-Al-Ti MPEAs. Six representative machine learning models were comprehensively benchmarked to identify the most effective predictor for the high-dimensional compositional space. SHAP (SHapley Additive exPlanations) analysis was further employed to interpret the intrinsic relationships between individual features and the target properties. To validate the model’s predictive capability, candidate MPEAs suggested by the model were fabricated and experimentally tested. The measured properties were compared with predictions to assess the model’s effectiveness and practical reliability. This integrated workflow not only provides a scientific foundation for designing MPEAs but also underscores the significant potential of ML for performance prediction in this emerging alloy class.

## 2. Methodology

### 2.1. Database and ML Model

**Figure 1:**
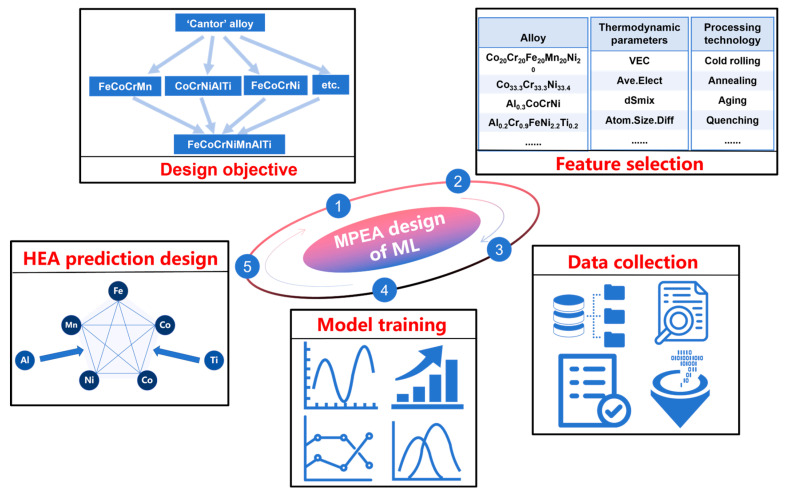
Schematic Diagram of Machine Learning-Assisted Fe-Co-Cr-Ni-Mn-Al-Ti MPEA Design.

Figure 1 schematically illustrates the ML-based design workflow for Fe-Co-Cr-Ni-Mn-Al-Ti MPEAs. The workflow begins with the dual requirements of the alloy system and its intended service conditions, focusing on an MPEA system derived from the FeCoCrNiMn “Cantor” base with Al and Ti additions for strengthening. By systematically reviewing the existing literature and considering engineering demands for combined strength and ductility, the design space and target property window for Fe-Co-Cr-Ni-Mn-Al-Ti alloys were established. Within this framework, an initial pool of candidate alloys was constructed based on elemental stoichiometry, and their compositions were mapped into feature vectors suitable for ML algorithms.

Feature selection serves as the critical link between data collection and model construction, directly governing the model’s predictive accuracy and generalization capability. Given the complex composition–microstructure–property relationships in MPEAs, input features were constructed from three interpretable categories. This approach avoids the “curse of dimensionality” and overfitting risks associated with purely data-driven feature searches while ensuring model outputs can be traced to specific physical quantities, which is essential for elucidating the mechanisms governing strength–ductility synergy.

First, elemental contents (in atomic percent, at.%) serve as the fundamental descriptors, directly influencing the average electronic structure, lattice parameter, and solid-solution strengthening. For the FeCoCrNiMn (Al, Ti) system, subtle variations in composition can markedly alter phase fractions and partitioning between the solid-solution matrix and secondary phases, thereby affecting mechanisms such as solid-solution strengthening, precipitation hardening, and transformation-induced plasticity. For instance, increased Al and Ti content promotes B2 or L1_2_ ordered phases, enhancing yield strength but potentially compromising ductility, while tuning Mn and Ni modifies stacking fault energy, influencing dislocation slip modes and deformation twinning. Using elemental content as primary features ensures model predictions are grounded in the actual influence of composition on underlying micromechanisms.

Second, thermodynamic descriptors related to phase formation and stability—including valence electron concentration (VEC), Average Electronegativity (Ave.Elect), atomic size mismatch (δ), and configurational mixing entropy (ΔSmix)—were constructed as higher-order features derived from composition. VEC influences the tendency to form FCC or BCC/B2 structures, thereby affecting active slip systems and matrix plasticity. Average electronegativity reflects the overall electron affinity of an alloy and the chemical bonding properties between atoms. Atomic size mismatch quantifies lattice distortion, directly controlling solid-solution strengthening and influencing dislocation mobility. ΔSmix captures the thermodynamic driving force for single-phase solid-solution formation. Incorporating these parameters distills physically meaningful quantities sensitive to phase constitution, mitigates redundancy from highly correlated raw variables, and enhances model interpretability.

Third, processing parameters associated with the fabrication route bridge “composition and thermodynamic potential” to the final microstructure. Homogenization temperature and time determine the extent of segregation removal and initial grain size. Cold-rolling reduction controls dislocation density, texture, and deformation band distribution, tailoring nucleation conditions for subsequent recovery, recrystallization, and precipitation. Solution and aging treatments, along with quenching strategy, dictate the morphology, size, and distribution of secondary phases, as well as potential phase transformations. The interplay of these parameters manifests in combined microstructural effects—grain refinement, dislocation structure evolution, precipitation strengthening, and transformation-induced plasticity—which collectively determine yield strength, uniform elongation, and TE. Including processing parameters as features enables the model to capture the critical reality that identical compositions can exhibit vastly different properties under different processing histories, granting the model practical relevance for guiding processing routes.

To eliminate human bias caused by ordinal coding and introduce clear physical meaning, this study replaces the categorical variable ‘cooling method’ with the continuous variable ‘estimated cooling rate ‘. Based on simulation and measured data of metal samples of the same size (approximately 2 mm thick) from heat transfer literature [36], the cooling rates corresponding to each process are assigned as follows:

- Furnace Cooling: 0.1 K/s. At this point, the sample is in a quasi-equilibrium state, and atoms diffuse sufficiently.
- Air Cooling: 5.0 K/s. Convection cooling, partially suppressing diffusion.
- Liquid Nitrogen Quenching: 40.0 K/s. Although the final temperature is low, due to the film barrier effect caused by the Leidenfrost effect, its high-temperature cooling rate is lower than that of water quenching [37].
- Water Quenching: 500.0 K/s. The nuclear boiling mechanism is dominant, exhibiting the highest quenching intensity and maximizing the retention of supersaturated vacancies and solid-solution atoms.

In summary, compositional, thermodynamic, and processing features jointly describe the hierarchical “alloy chemistry–phase stability–microstructural evolution” chain. Fixing these three feature categories ensures the input space carries clear physical meaning and generality, facilitating transfer to new alloy systems. Model predictions can be decomposed along specific feature dimensions for sensitivity analysis, enabling quantitative assessment of the marginal contribution of each element, thermodynamic parameter, and processing step to the strength–ductility balance. This provides an interpretable basis for subsequent compositional optimization and process design. Original compositional data, thermodynamic parameters, and processing parameters were converted into numerical features following the equations in Table 1.

This study did not model yield strength because the definitions of yield strength values reported in the literature are often inconsistent (e.g., 0.2% offset versus upper/lower yield strength), and the missing rate for yield strength was much higher than for tensile strength and total elastic modulus in the selected records. To maintain data consistency, we focused primarily on UTS and TE.

A comprehensive experimental dataset for Fe-Co-Cr-Ni-Mn-Al-Ti MPEAs was compiled based on the established feature system. Initial screening of open literature yielded over 850 records with explicit compositions, heat-treatment parameters, and room-temperature mechanical properties. To ensure consistency and reliability, records with incomplete information, missing parameters, or non-standard testing conditions were removed. Entries suspected of containing typographical errors or exhibiting outliers inconsistent with global trends were carefully examined and discarded when necessary. Ultimately, 671 high-quality data points were retained to construct the final database. Each point corresponds to a complete sample vector coupling “composition–processing–thermodynamic features–strength–ductility metrics,” providing a robust foundation for model training.

This database was established by screening peer-reviewed studies reporting the room-temperature tensile properties of Fe-Co-Cr-Ni-Mn-Al-Ti (based) alloys. The literature was searched in the Web of Science database using keyword combinations including “high-entropy alloys”, “multi-principal element alloys”, “tensile”, “ultimate tensile strength”, “elongation”, “annealing”, and “aging”. The literature was reviewed only if it: (i) explicitly reported alloy composition (atomic percentage); (ii) had a complete processing history (including at least homogenization/rolling/annealing/aging and cooling methods, if applicable); and (iii) explicitly reported room-temperature ultimate tensile strength and elongation or included tensile curves. Data points lacking key processing variables, reported under non-standard testing conditions, or with significant inconsistencies in units or composition were excluded. All units were standardized, and duplicate records were removed. Table S1 provides a list of references for each included data point and its source.

To obtain a reliable predictive model with a limited sample size, a classical train-test split strategy was adopted. The 671 samples were randomly divided into a training set (538 samples, 80%) for model fitting and hyperparameter optimization, and an independent test set (133 samples, 20%) to assess the final model’s generalization capability and true predictive performance. A fixed random seed was used for partitioning to ensure reproducibility.

Figure 1 further outlines the complete ML pipeline from raw data to trained model. Original compositional data, thermodynamic parameters, and processing parameters were converted into numerical features following the equations in Table 1. Features with different physical dimensions were standardized to eliminate scale-disparity effects on model training. Several mainstream regression algorithms were then constructed and compared within the same feature space and under identical data partitions: Random Forest Regressor, Support Vector Regression (SVR), Decision Tree Regressor, fully connected feed-forward Neural Network Regressor, Gradient Boosting Decision Tree (GBDT), and eXtreme Gradient Boosting (XGBoost, version 3.0.4) Regressor. All models were trained and evaluated under consistent conditions to ensure direct performance comparability.

To minimize overfitting and fully utilize the limited data, leave-one-out cross-validation (LOOCV) was applied to the training set. In this scheme, each sample in the training set is used once as a validation sample while the remaining samples form the training subset. The cross-validation error obtained provides a robust estimate of model performance on unseen data and guides hyperparameter tuning and model selection. LOOCV offers higher data utilization and more stable evaluation in small-sample regimes compared to simple single-split validation.

Model performance was quantitatively evaluated using complementary metrics. The mean absolute error (MAE) measures the average deviation between predicted and experimental values:

(1) $$MAE=\frac{1}{n}\sum_{i=1}^{n}|y_{i}-{\hat{y}}_{i}|$$

where $y_{i}$ i is the experimental value for the i-th sample, ${\;ŷ}_{i}$ is the corresponding prediction, and n is the total number of samples.

The root mean square error (RMSE) assigns greater weight to large deviations:

(2) $$RMSE=\sqrt{\frac{1}{n}\sum_{i=1}^{n}(y_{i}-{\hat{y}}_{i}{)}^{2}}$$

The coefficient of determination (R^2^) assesses the proportion of variance explained by the model:

(3) $$R^{2}=1-\frac{\sum_{i=1}^{n}(y_{i}-{\hat{y}}_{i}{)}^{2}}{\sum_{i=1}^{n}(y_{i}-\overset{-}{y}{)}^{2}}$$

where ȳ is the mean of all experimental values. An excellent model should exhibit low MAE and RMSE alongside an R^2^ value close to 1. By systematically comparing these metrics across models, the optimal model for predicting the strength–ductility behavior of Fe-Co-Cr-Ni-Mn-Al-Ti MPEAs was objectively identified.

After selecting the optimal model, feature importance analysis was conducted to elucidate the contributions and sensitivities of each elemental content, thermodynamic parameter, and processing variable to the strength–ductility response. The extent of solidification segregation is not explicitly quantified in the present descriptor set; instead, homogenization temperature and time are used as proxy variables reflecting the degree of chemical homogenization prior to deformation and heat treatment. This analysis facilitates the extraction of design principles for performance optimization. Finally, targeted experimental fabrication and mechanical testing of high-performance candidate alloys proposed by the model were performed to validate the ML predictions, establishing a closed loop from data-driven design to experimental verification.

This study defines the target window as achieving ultra-high strength (UTS ≥ 1600 MPa) while maintaining practically usable ductility (TE ≥ 10%). This criterion was chosen based on the reported distribution of Fe-Co-Cr-Ni-Mn-Al-Ti (based) alloys in our collected dataset, and the necessity of avoiding premature brittle fracture in ultra-high strength candidate materials.

All models were implemented in Python (version 3.12). The XGBoost regressor was built using the XGBoost package (version 3.0.4), and baseline models were implemented using scikit-learn (version 1.5.1). SHAP analyses were performed using the SHAP package (version 0.48.0). Data processing and visualization were conducted with numpy (versions 1.26.4), pandas (versions 2.2.2), and matplotlib (versions 3.9.2).

### 2.2. Experimental Validation

The target MPEA compositions were prepared by vacuum arc melting high-purity elemental feedstocks (>99.9 at.%) under an argon atmosphere. Arc melting was conducted under a high-purity Ar atmosphere after evacuating the chamber to 8.0 × 10^−4^. Prior to melting, the elemental feedstocks were mechanically cleaned to remove surface oxides, and a Ti getter melt was employed to further reduce residual oxygen. To minimize volatilization and oxidation during multiple remelts, the melting time per cycle was kept constant 5 min and the ingot was flipped between cycles to improve chemical homogeneity. The standard laboratory VAR (Vacuum-Based Arrival) procedure involves remelting 4–6 times. To strike a balance between chemical homogenization and minimizing elemental loss, six remelts were employed. Beyond this number, additional remelting offers only limited improvement in compositional uniformity while increasing the risk of oxidation/volatilization, particularly for Al and Ti. Melt into ingots measuring 80 × 35 × 10 mm. The as-cast ingots were homogenized at 1200 °C for 2 h under argon to eliminate solidification segregation, followed by furnace cooling. The homogenized plates were cold-rolled along the longitudinal direction to approximately 80% thickness reduction, with a per-pass reduction of 0.2 mm. Subsequently, the cold-rolled samples were annealed at 700 °C for 1 h (designated condition: 700-1). A portion of the 700-1 samples were further aged at 550 °C for 24 h (designated condition: 700-1-550-24). All heat-treated specimens were water-quenched to retain the microstructures formed at the respective temperatures.

Crystal structure and phase constitution were characterized by X-ray diffraction (XRD, D/Max-2500/PCX, Rigaku Corporation, Tokyo, Japan) using Cu Kα radiation, with a scanning range of 20–100° at a rate of 4°/min. Sample surfaces were mechanically polished prior to testing to ensure data accuracy. Microstructural observations and elemental distribution analyses were performed using a field-emission scanning electron microscope (FE-SEM, JEOL JIB-4700F, JEOL Ltd., Tokyo, Japan) equipped with energy-dispersive X-ray spectroscopy (EDS) for elemental mapping.

Dog-bone-shaped tensile specimens were machined from the rolled plates via electrical discharge machining (EDM), with a gauge length of 8 mm and a cross-sectional width of approximately 1.3 mm along the rolling direction. Specimen surfaces were ground with SiC abrasive papers to remove oxide layers prior to testing. Room-temperature uniaxial tensile tests were conducted on a universal testing machine (Instron 5982, Illinois Tool Works Inc, Norwood, MA, USA) at a constant strain rate of 1 × 10^−3^ s^−1^. An extensometer was used to monitor strain during deformation. To ensure reproducibility, tensile tests for each heat-treatment condition were performed in triplicate.

## 3. Results

### 3.1. Performance Evaluation of Machine Learning Models

**Figure 2:**
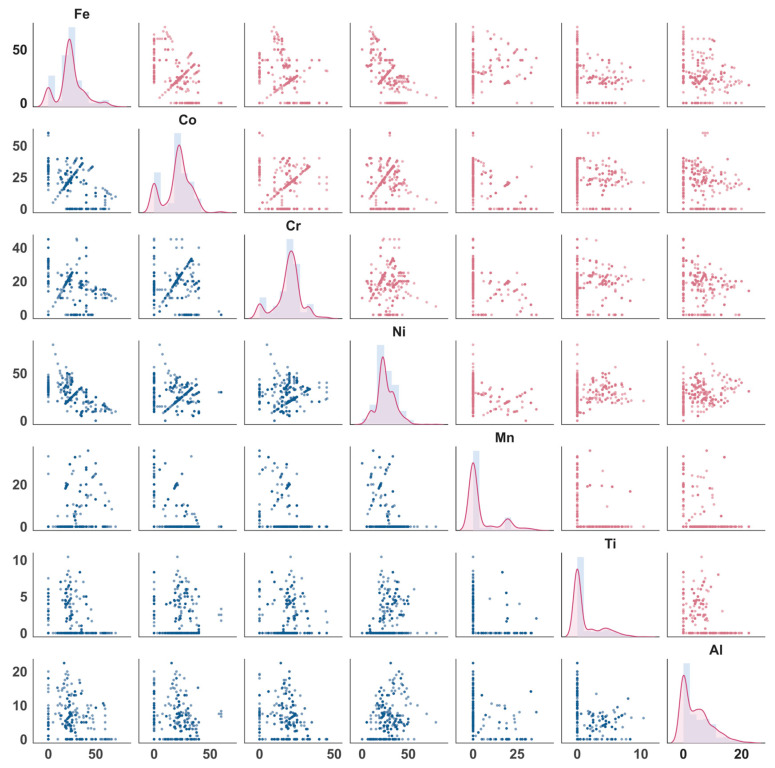
Scatter matrix of the elemental contents in the Fe-Co-Cr-Ni-Mn-Al-Ti MPEA dataset.

To assess the validity of the constructed feature space and data distribution, a visual analysis was first performed on the atomic content of the seven constituent elements (Fe, Co, Cr, Ni, Mn, Al, Ti), as shown in Figure 2. The figure presents a compositional scatter matrix: the diagonal plots display the probability distribution for each element’s atomic fraction, while the off-diagonal plots illustrate the bivariate relationships between any two elements. The base elements (Fe, Co, Cr, Ni, Mn) exhibit a broad distribution ranging from 0 to 50 at.%, encompassing both near-equiatomic compositions and those with significant deviations (enriched or depleted). This indicates that the dataset covers a wide compositional window, from typical “Cantor-type” alloys to those with pronounced elemental partitioning. In contrast, the contents of Al and Ti are notably concentrated within lower ranges (0–10 at.% and 0–20 at.%, respectively), with a significant number of samples containing zero Al or Ti. This reflects their primary role as minor strengthening additions rather than principal constituents. The distribution pattern of “wide-ranging base elements + low-content strengtheners” aligns with the subsequent constraint applied in the inverse design, where the combined content of Al and Ti does not exceed 20 at.%, ensuring the model’s application scope remains physically consistent with the training data. Several element pairs in the scatter matrix, such as Al–Ti, Al–Mn, and Ti–Mn, show notable negative correlations or “L”-shaped distributions. This indicates that the addition of strengthening elements is often compensated by adjustments to other components, a potential source of collinearity that must be considered during ML modeling.

**Figure 3:**
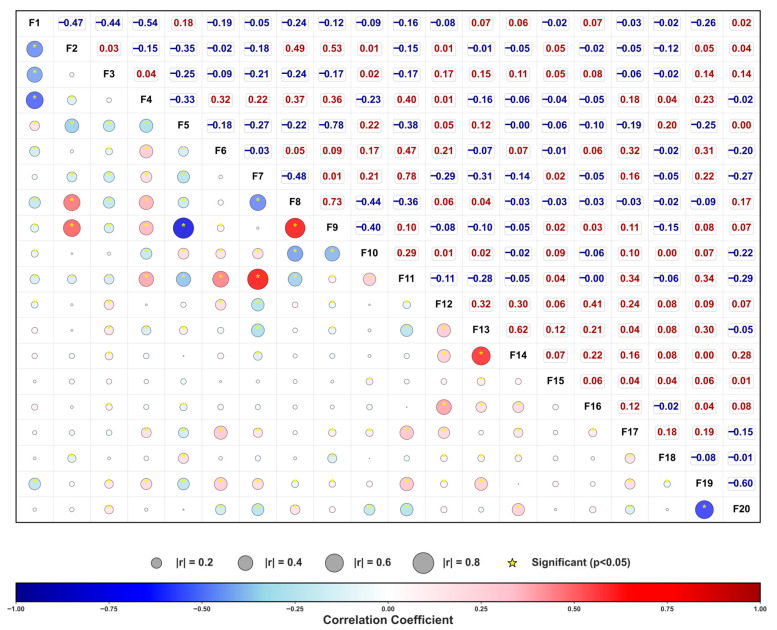
Bubble matrix diagram of the correlation among various characteristic parameters. This figure shows the pairwise correlations among 18 characteristic parameters from F1 to F18, which are quantitatively analyzed using the Pearson correlation coefficient. The horizontal and vertical axes are both feature numbers F1–F20, and their physical meanings correspond as follows: F1: Fe, F2: Co, F3: Cr, F4: Ni, F5: Mn, F6: Ti, F7: Al, F8: VEC, F9: Ave.Elect, F10: dSmix, F11: Atom.sides.diff, F12: Hom_Temp (°C), F13: Rolling reduction, F14: Anneal_Temp (°C), F15: Anneal_Time (h), F16: Quench, F17: Age_Temp (°C), F18: Age_Time (h), F19: UTS (MPa), F20: TE (%). Each bubble in the figure represents the strength and positivity of the correlation between the corresponding two variables. Color indicates the positive or negative value and magnitude of the correlation coefficient. The color scale range is from −1 to +1, where blue represents negative correlation and red represents positive correlation. The darker the color, the greater the absolute value of the correlation. The size of the bubble is directly proportional to the absolute value of the correlation coefficient (r). The legend provides reference scales of r = 0.2, 0.4, 0.6, and 0.8 for visually comparing the strength of the correlation between different variable pairs. The values are marked as the corresponding correlation coefficient r, facilitating precise comparison and quantitative analysis. The yellow star indicates that the correlation is statistically significant (*p* < 0.05), suggesting that the linear correlation between the variables is statistically reliable.

Building on this, the Pearson correlation coefficients between all 18 features (elemental contents, thermodynamic descriptors, and process parameters) and the two target mechanical properties were calculated and visualized in a bubble chart (Figure 3). A significant negative correlation (Pearson r ≈ −0.6) exists between the two targets, illustrating the classic strength–ductility trade-off. This aligns with the established understanding in both conventional and MPEAs that strengthening mechanisms frequently compromise ductility. From a compositional perspective, a strong positive correlation is observed between (Al, Ti) content and the atomic size mismatch parameter (δ), as the larger atomic radii of Al and Ti relative to other transition metals induce substantial lattice distortion. This facilitates strength enhancement via solid-solution hardening and ordered phase precipitation but may concurrently hinder dislocation glide coordination, thereby reducing ductility. Among process-related features, a strong positive correlation exists between rolling reduction and annealing temperature. This trend, prevalent in the existing experimental data, indicates that high-deformation cold rolling is often coupled with higher-temperature recrystallization annealing to restore plasticity lost to work hardening—a common practice in industrial processing. It is crucial to emphasize that the Pearson coefficient only captures linear relationships. Many near-zero correlations in Figure 3 do not imply these variables are irrelevant to strength–ductility behavior but may instead reflect threshold effects or highly nonlinear responses. Furthermore, some seemingly significant correlations might be constrained by confounding variables or experimental design biases. Therefore, Figure 2 and Figure 3 are more valuable for verifying data distribution rationality, identifying significant collinearity, and uncovering potential couplings, rather than providing sufficient conditions for alloy design. It is precisely this inherent nonlinearity and high-dimensional coupling that necessitates the use of machine learning models.

**Figure 4:**
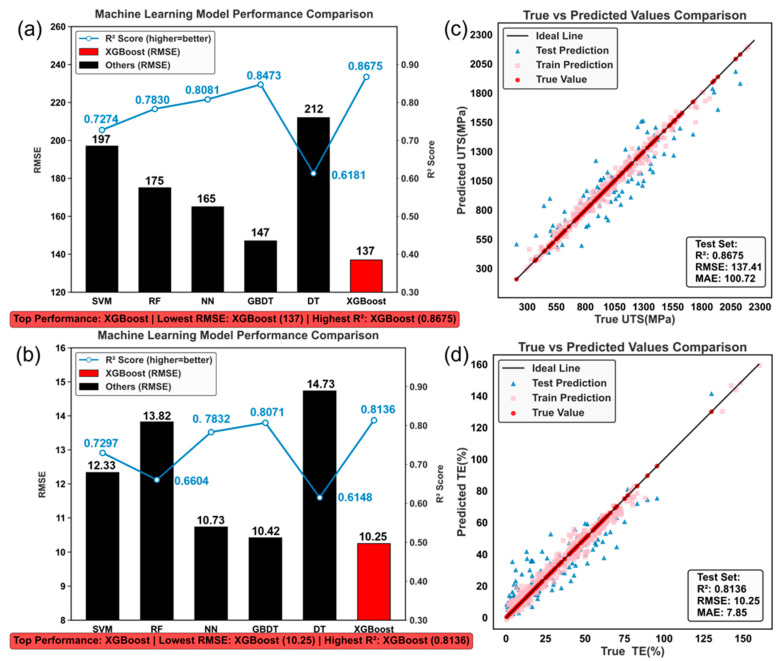
Performance evaluation of different machine learning models and comparison of the optimal model’s predictions with actual data. The figure uses a compact layout: (**a**) UTS and (**b**) TE are juxtaposed as two subplots, visually presenting the R^2^ (coefficient of determination; higher is better) and RMSE (root mean square error) evaluation results for the two target properties. Among the models, XGBoost demonstrates the best performance for predicting both properties. The evaluation metrics for UTS prediction are superior to those for TE prediction. Subplots (**c**,**d**) depict the actual-predicted value scatter distributions for UTS and TE, respectively, with the diagonal line representing the ideal reference. For UTS prediction, the model achieves higher accuracy on the test set, and the prediction error is more uniform across different strength levels. For TE prediction, the test data also shows high accuracy; however, the prediction error is higher for low elongation values.

The core objective of this study is to utilize the compiled Fe-Co-Cr-Ni-Mn-Al-Ti MPEA dataset to train and select an optimal ML model for high-accuracy prediction of UTS and elongation for alloys with unknown compositions and varying processing conditions. To this end, six regression models were constructed based on the unified 18-dimensional feature space, and their predictive performance was systematically compared using identical training/test splits and cross-validation strategies. Figure 4 summarizes the R^2^ and RMSE metrics for the different models in predicting UTS and elongation, alongside the true-vs.-predicted scatter plots for the best-performing models. For UTS (Figure 4a), XGBoost and GBDT significantly outperformed other classical models. XGBoost, with its regularized objective function, second-order gradient optimization, and efficient splitting algorithm, strikes an effective balance between preventing overfitting and enhancing generalization capability. It achieved an R^2^ of 0.8675 and an RMSE of ~137 MPa on the test set, and was therefore selected as the core model for subsequent analysis and inverse design. For fracture elongation (Figure 4b), XGBoost again delivered the best performance with an R^2^ of 0.8136, but the RMSE was notably higher at ~10.03%. This reflects the higher sensitivity of ductility to microstructural details and local deformation compatibility, suggesting elongation exhibits greater intrinsic randomness and experimental scatter, posing a more stringent challenge for data-driven models.

The true vs. predicted scatter plots further illustrate the model’s predictive behavior. For UTS (Figure 4c), predicted points cluster tightly along the ideal diagonal, with training and test sets nearly overlapping, indicating XGBoost does not exhibit obvious overfitting. The error distribution is relatively uniform across different strength levels, demonstrating good accuracy for both high- and medium-strength alloys. In contrast, the data points for total elongation (TE, Figure 4d) show greater scatter, with notable prediction errors in the low-ductility region (TE < 10%). Some samples exhibit systematic over- or under-prediction, reflecting the fact that ductility is influenced by multiscale factors—such as local defects, second-phase morphology, and strain localization—many of which are not fully captured in the current 18-dimensional feature space. Overall, based on the performance metrics in Figure 4, XGBoost was selected as the optimal model for subsequent property prediction, feature importance analysis, and inverse design.

Following model selection, XGBoost was employed for the inverse prediction and screening of MPEAs across different compositions and processing routes. The objective was to design high-strength alloys with acceptable ductility while rigorously verifying the model’s reliability. Here, ‘acceptable plasticity’ is defined as a total elongation (TE) greater than 10%. This threshold references the general safety margin requirements for ultra-high strength structural materials (such as maraging steel and high-strength titanium alloys) in the aerospace field [38]. Given the high-dimensional, continuous input space and computational constraints precluding an exhaustive search, the design space was reasonably constrained based on physical principles and data distribution. Compositionally, the contents of the five base elements (Fe, Co, Cr, Ni, Mn) were limited to 0–50 at.% with a discrete step of 5 at.%. Al and Ti, as strengtheners, were constrained to a combined content of 0–20 at.% to avoid extrapolating beyond the empirical window of the database, ensuring their role as minor additions for precipitation hardening and lattice distortion. Reference [34] illustrates that with an appropriate Al/Ti ratio and proper heat treatment (rapid cooling + aging), fine and dispersed coherent L1_2_ phases can be precipitated, achieving a strong-plastic synergy, rather than coarse brittle phases leading to brittle fracture. The total elemental content summed strictly to 100 at.%, with each alloy containing at least four elements to maintain its high- or medium-entropy solid-solution character. Regarding processing, homogenization was fixed at 1200 °C for 2 h, a common practice in the literature to eliminate macrosegregation and dendritic structures in cast MPEAs. Cold-rolling reduction was set to 80% to introduce sufficient dislocation density and stored energy, driving subsequent recrystallization and precipitation [39,40,41,42]. Annealing and aging parameters were varied to control grain size and second-phase precipitation: annealing temperature was set between 800–1100 °C for 0–4 h to govern recrystallization and grain refinement; aging temperature was limited to 400–750 °C for 0–100 h to promote fine precipitation while avoiding brittle phase formation. The cooling method was uniformly set to water quenching to rapidly “freeze” the high-temperature microstructure and prevent uncontrolled phase transformations. These constraints reflect both current experimental windows and serve to minimize model extrapolation beyond the training data, enhancing prediction reliability. Within this constrained design space, XGBoost was used to batch-predict properties for approximately 1000 MPEA composition–process combinations. Two alloys with identical composition but different heat-treatment paths and superior predicted performance were selected for experimental validation. Their specific compositions and processing parameters are listed in Table 2. The cold-rolled Co_30_Cr_15_Ni_45_Al_5_Ti_5_ alloy, annealed at 700 °C for 1 h, is designated “700-1”; a subsequent aging at 550 °C for 24 h yields the “700-1-550-24” condition. Comparing these two alloys will not only test the model’s sensitivity to heat treatment but also aid in understanding the underlying strengthening mechanisms from a microstructural perspective. Although the search was conducted within the Fe-Co-Cr-Ni-Mn-Al-Ti composition space, the optimal candidate materials determined under given constraints lie in the Co-Cr-Ni-Al-Ti subset (where the contents of Fe and Mn are close to zero). This does not imply that Fe or Mn is generally harmful; rather, it reflects the optimal solution for the current objective and data domain.

Although the two processing routes yield relatively close predicted properties, they were intentionally selected from a near-optimal region to examine the model’s sensitivity to subtle heat-treatment variations. A broader in silico sweep of aging parameters for the same composition is provided in Figure S1, showing that the model captures the overall strengthening trend with aging while the predicted ductility response remains less sensitive, consistent with the intrinsic scatter of TE in the literature data.

### 3.2. SHAP Analysis

**Figure 5:**
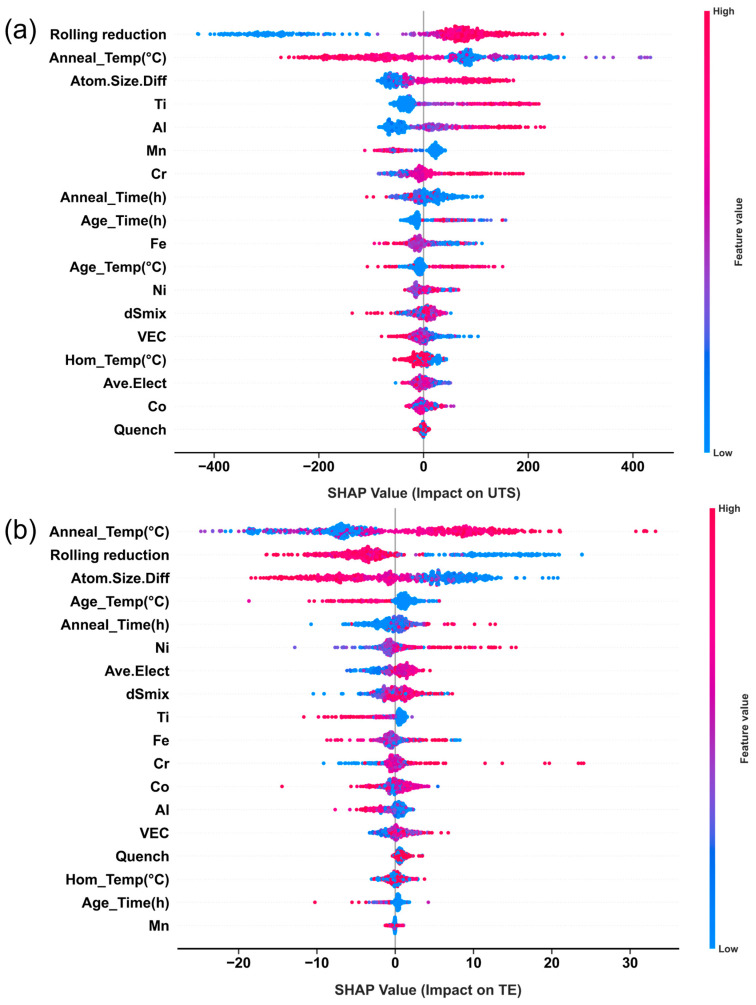
SHAP contribution analysis of feature importance. (**a**) SHAP analysis for UTS; (**b**) SHAP analysis for TE.

To elucidate the contribution mechanisms of each input feature to the strength–ductility behavior, a systematic SHAP (SHapley Additive exPlanations) analysis was performed on the optimal XGBoost model. The SHAP method quantifies the marginal contribution of each feature to model predictions at the sample level and provides a global ranking of feature importance. Figure 5 presents the global SHAP summary plots for UTS and TE. For UTS, rolling reduction and annealing temperature exhibit the most significant SHAP values with opposing signs: rolling reduction shows a strong positive contribution, whereas annealing temperature generally shows a negative one. This indicates that dislocations and substructures introduced by plastic deformation are primary sources of strength, while high-temperature annealing weakens this contribution via recrystallization and recovery. Elements such as Cr, Al, and Ti display consistently positive SHAP values, suggesting their appropriate addition significantly enhances UTS through solid-solution distortion and ordered-phase precipitation. Thermodynamic descriptors like electronegativity difference (Δχ) and atomic size mismatch (δ) also show notable positive contributions, further confirming that “chemical short-range order + lattice distortion” is a key physical basis for high-strength design in this system. In contrast, for TE, the influence of process parameters is more pronounced: judicious combinations of annealing temperature/time and rapid cooling (e.g., water quenching) provide the most positive contributions to ductility, while prolonged aging and high δ values exhibit significant negative contributions, pointing to precipitate coarsening and early cracking induced by excessive lattice distortion. Notably, some elements (e.g., Cr) maintain consistent SHAP sign for both UTS and TE, indicating their potential for synergistic enhancement of strength and ductility. Conversely, other factors (e.g., Al content, aging time) exhibit a “positive-negative reversal,” revealing the inherent trade-off in compositional and process design.

SHAP values quantify the contribution of each feature to an individual prediction based on Shapley values from cooperative game theory. For a given sample, the model output can be written as $f(x)=\phi_{0}+\sum_{i=1}^{M}\phi_{i}$, where $\phi_{0}$ is the expected model output and $\phi_{i}$ is the contribution of feature i. For tree-based models, SHAP values were computed using the TreeSHAP algorithm. Global feature importance was assessed by the mean absolute SHAP value, $mean(|\phi_{i}|)$, across all samples.

**Figure 6:**
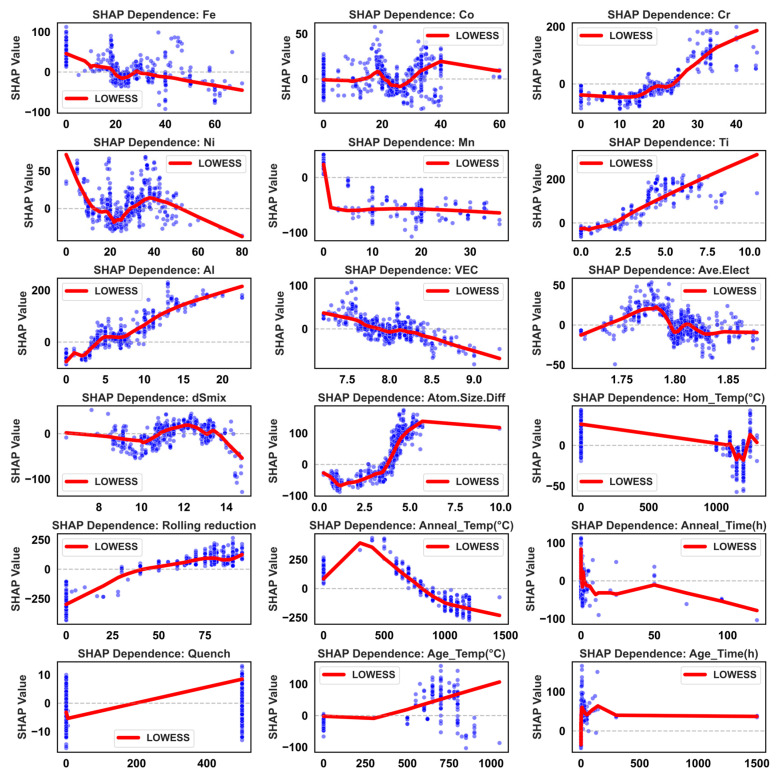
Scatter plots of UTS versus features. *Y*-axis: Represents the SHAP value, which quantifies the feature’s impact on the model output. Red line: Denotes the LOWESS (Locally Weighted Scatterplot Smoothing) fit, illustrating the general trend between the feature and its SHAP value.

**Figure 7:**
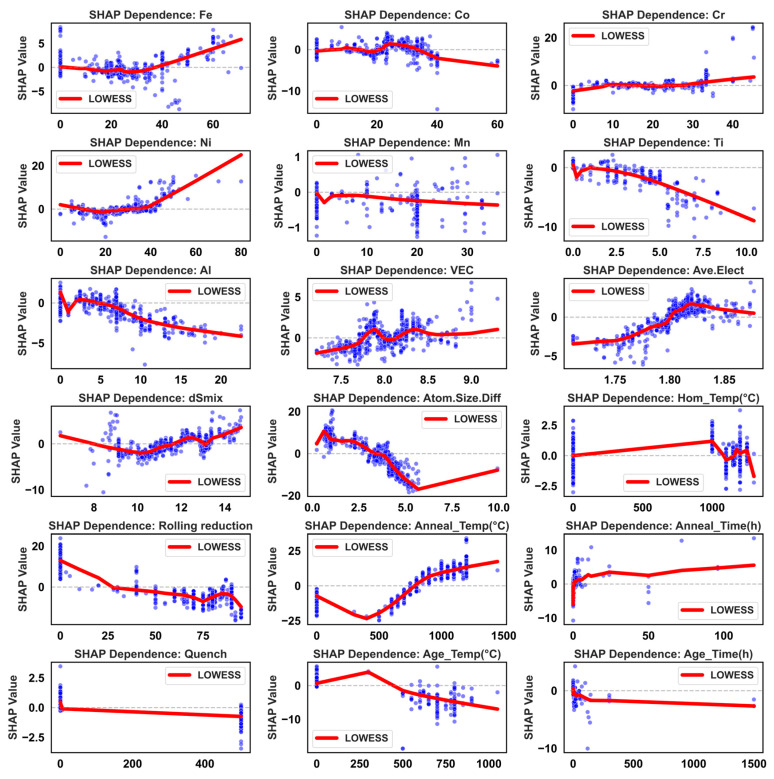
Scatter plots of TE versus features. *X*-axis: Represents the feature value. *Y*-axis: Represents the SHAP value, which quantifies the feature’s impact on the model output. Red line: Denotes the LOWESS (Locally Weighted Scatterplot Smoothing) fit, illustrating the general trend between the feature and its SHAP value.

To obtain more physically insightful quantitative relationships, SHAP dependence scatter plots for key features against UTS and TE are presented in Figure 6 and Figure 7, respectively, with overall trends smoothed using LOWESS. For UTS (Figure 6), most elemental contents show approximately monotonic or piecewise-monotonic relationships with SHAP values: Fe content has a limited influence at low-to-moderate levels but becomes significantly negative beyond ~30 at.%, likely due to precipitation of brittle σ-phase. Ni content shows a turning point near 40 at.%, beyond which further increases may coarsen the FCC matrix and reduce grain boundary strengthening, thereby weakening strength. Co content exhibits a “dip” in the 20–40 at.% range, suggesting excessive Co may reduce stacking fault energy, promoting twinning or ε-martensite formation and altering the fracture mode. In contrast, Cr, Al, and Ti show consistently positive SHAP trends over broad content ranges, highlighting their crucial role in solid-solution and precipitation strengthening. However, SHAP values drop sharply when Cr content falls below ~10 at.%, indicating insufficient Cr may fail to stabilize favorable multiphase microstructures and could induce brittleness. Thermodynamic descriptors show that increasing VEC correlates with decreasing UTS, suggesting compositions with lower VEC and a tendency toward BCC/B2 structures favor higher strength. Electronegativity difference and mixing entropy provide positive contributions within certain ranges, while increasing atomic size mismatch boosts SHAP values, further confirming the role of pronounced lattice distortion in high-strength MPEA design. Process parameters, including rolling reduction, annealing temperature/time, and quenching method, regulate UTS by affecting grain size, dislocation density, and second-phase morphology at the microstructural level.

For TE (Figure 7), the feature–SHAP relationships are more nonlinear and asymmetric, reflecting the high sensitivity of ductility to microstructural homogeneity and deformation compatibility. VEC exhibits a characteristic inverted “bell-shaped” curve for TE, peaking near 10.5, which corresponds to the electronic structure window where FCC/TWIP or TRIP mechanisms can most effectively collaborate. The parameter δ shows a continuous negative contribution across its entire range, particularly when δ > 4, with ~95% of data points falling in the negative SHAP region. This indicates that excessive lattice distortion, while beneficial for strength, significantly impairs ductility. Ni content and moderate annealing temperatures (~800–1100 °C) are the two core factors for improving TE, with SHAP peaks exceeding 20. This aligns with Ni’s role in stabilizing the FCC structure and the ability of moderate annealing to promote fine, uniform recrystallization. In contrast, higher Al content and slow cooling (e.g., furnace cooling) correspond to significant negative SHAP values, revealing that coarse ordered phases and microstructural coarsening from long-range diffusion are key sources of plasticity loss. Moderate values of average atomic size and intermediate rolling reduction exhibit “optimal window” characteristics, suggesting that maintaining sufficient deformation energy while avoiding excessive work hardening and texture concentration is key to balancing properties. High homogenization temperatures (>1200 °C) form an independent positive SHAP cluster for TE, reflecting the benefit of eliminating coarse dendrites and macrosegregation. However, aging times exceeding ~24 h generally yield negative SHAP values, attributable to precipitate coarsening, weakened interfaces, and potential formation of new brittle phases.

Collectively, Figure 2, Figure 3, Figure 4, Figure 5, Figure 6 and Figure 7 demonstrate that the constructed feature space not only statistically covers the primary composition and processing windows of Fe-Co-Cr-Ni-Mn-Al-Ti alloys but also, through the XGBoost-SHAP framework, provides an interpretable mapping from “composition–thermodynamics–processing” to “strength–ductility.” Linear correlation analysis reveals macro-level trade-offs and feature co-evolution, while the high R^2^ and low RMSE of the XGBoost model for both UTS and TE demonstrate its capability for reliable performance prediction in complex MPEA systems. The SHAP results further quantify the marginal contributions of each element, thermodynamic descriptor, and processing parameter, providing a physically grounded, quantitative basis for the subsequent design and rapid screening of MPEAs across broader compositional and processing spaces.

## 4. Discussion

**Figure 8:**
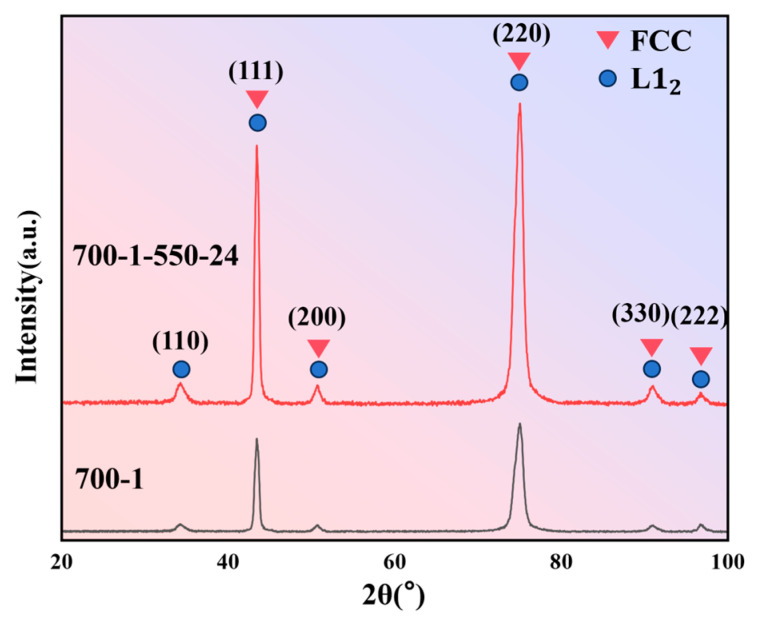
XRD results of 700-1 and 700-1-550-24.

Figure 8 presents the XRD patterns of the two inversely designed MPEAs under different heat-treatment conditions. The diffraction peaks for both states can be indexed to an FCC matrix alongside minor L1_2_ ordered-phase reflections. This indicates that the Co_30_Cr_15_Ni_45_Al_5_Ti_5_ alloy forms a typical duplex microstructure consisting of an FCC solid-solution matrix with dispersed L1_2_ precipitates after short-term annealing at 700 °C. Following aging at 550 °C for 24 h, the L1_2_ superlattice peaks (e.g., (110), (210), (211)) intensify and sharpen, implying an increase in both the volume fraction and coherent size of the ordered precipitates. This microstructural evolution aligns closely with the SHAP importance analysis in Section 3.2, where Al and Ti contents, as well as aging parameters, were identified as making significant positive contributions to UTS but negative contributions to ductility—a direct consequence of the precipitation and coarsening of L1_2_ phases. The XRD patterns show an FCC-dominated microstructure, which can be attributed to the strong FCC-stabilizing effect of the high Ni content and the overall electronic structure of the designed composition. No distinct diffraction peaks associated with HCP-related phases or coarse intermetallic compounds were observed, consistent with an FCC matrix strengthened primarily by ordered L1_2_-type precipitates rather than by large-volume-fraction brittle phases.

**Figure 9:**
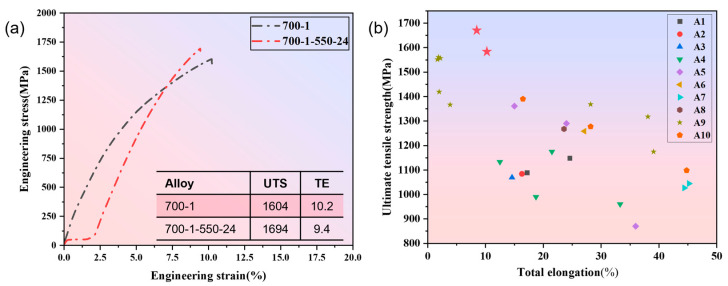
Tensile mechanical properties and performance comparison. (**a**) Tensile curve and property table. (**b**) Comparison of this work with other studies [43,44,45,46,47,48,49,50,51,52].

Figure 9 further details the tensile mechanical properties of the designed alloys and provides a comparison with literature data. For the Co_30_Cr_15_Ni_45_Al_5_Ti_5_ system, the engineering stress–strain curve in Figure 9a shows that the 700-1 condition (700 °C/1 h anneal) achieves an excellent strength–ductility synergy: an UTS of 1604 MPa and a TE of 10.2%. This alloy achieves the core objective of this design while meeting the general safety margin requirements for ultra-high strength structural materials. The pronounced continuous work-hardening stage suggests the retention of a high dislocation density and moderate nanoprecipitates within a uniform FCC matrix, facilitating a synergistic multi-stage deformation mechanism. After aging at 550 °C for 24 h (700-1-550-24 condition), the UTS increases further to 1694 MPa, while TE decreases slightly to 9.4%, exhibiting the classic trend of “increased strength at the expense of some ductility.” This experimental observation is in excellent agreement with the conclusions derived from the XGBoost–SHAP framework: aging time shows a continuous positive contribution to UTS in the SHAP plot but a significant negative effect on TE, reflecting the inherent trade-off between L1_2_ precipitation strengthening and associated embrittlement mechanisms. The literature comparison in Figure 9b highlights the performance advantage of the alloys from this study. The two conditions (700-1 and 700-1-550-24), marked by red stars, lie near the Pareto front in the UTS-TE property space. The comparison in Figure 9b is intended to demonstrate that, in the ultra-high strength range above 1600 MPa, our alloy did not undergo brittle fracture (TE < 2–3%), but instead maintained 10% of engineering-usable ductility, making it more promising for applications than high-strength but extremely brittle alloys such as some refractory HEAs or metallic glasses. Their overall performance surpasses most reference systems, which typically exhibit UTS < 1500 MPa or TE < 10%, demonstrating the practical feasibility and clear performance benefit of the ML-driven composition–processing design pathway for Fe-Co-Cr-Ni-Mn-Al-Ti MPEAs.

A quantitative assessment of the model’s predictive reliability was conducted. For the 700-1 condition, the model predicted a UTS of 1713 MPa, approximately 6.7% higher than the experimental value of 1604 MPa, while the predicted TE of ~32% significantly overestimated the measured 10.2%. This suggests that for a solid-solution and deformation-strengthened microstructure, the model overestimates the contribution of plasticity-enhancing mechanisms (e.g., nano-twinning, dislocation cell structures) and their effect on work-hardening rate. It indicates that the current 18-dimensional feature set does not fully capture the influence of fine-scale defect structures on local strain homogeneity. In contrast, for the 700-1-550-24 condition, the model’s UTS prediction (1695 MPa) matches the experimental result perfectly, demonstrating accurate quantification of the precipitation strengthening contribution. However, the TE prediction remains at ~22%, far exceeding the experimental 9.4%. This indicates that while the model successfully identifies the strengthening role of γ’/L1_2_ ordered phases, it underestimates the detrimental effects on ductility from precipitate coarsening and associated early cracking at grain boundaries or triple junctions. This discrepancy—accurate strength prediction but optimistic ductility prediction—corroborates the strong nonlinearity observed in the SHAP dependence plot for aging time, where SHAP values become predominantly negative at longer durations. It further illustrates that ductility is governed by multiscale microstructural inhomogeneity and damage evolution, a more complex phenomenon than strength. Importantly, the model’s prediction regarding the positive effect of rapid cooling (water quenching) is fully consistent with experimental results. Water quenching effectively suppresses the formation of high-temperature equilibrium phases and ‘freezes’ a supersaturated solid solution, enabling subsequent mid-temperature aging to produce fine, dispersed L1_2_ precipitates within a matrix of high dislocation and vacancy density. This result validates the design philosophy of leveraging “kinetic control over thermodynamic equilibrium”—optimizing the processing path to guide the microstructure into a metastable state favorable for strength–ductility synergy is a key strategy in MPEA design.

**Figure 10:**
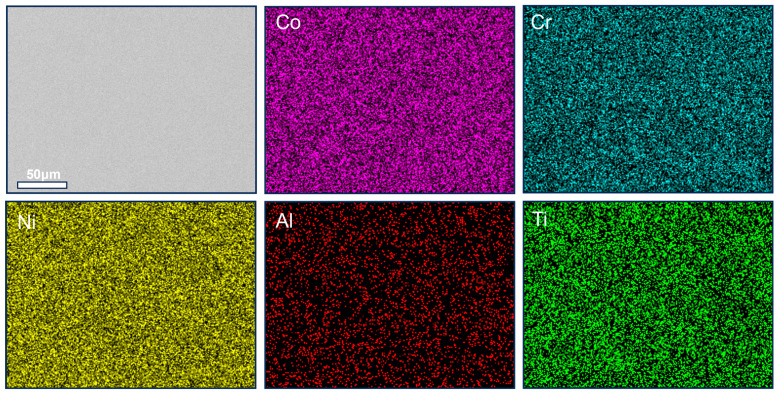
BSE image and corresponding EDS mapping of 700-1 MPEA.

Figure 10 presents EDS characterization results for the 700-1 condition. The BSE image reveals a uniform matrix devoid of second-phase particles larger than ~1 μm. Combined with elemental maps, this confirms that after annealing at 700 °C for 1 h, the Co_30_Cr_15_Ni_45_Al_5_Ti_5_ alloy forms a macroscopically homogeneous solid-solution structure. Elements Co, Cr, and Ni are uniformly distributed, forming continuous FCC solid-solution channels approximately 50 μm in width. This supports the earlier SHAP analysis where Ni showed a positive contribution to ductility, likely by stabilizing the single-phase FCC matrix and suppressing brittle σ-phase formation. Al and Ti are dispersed, with locally co-enriched regions having a characteristic size below ~350 nm. In these Al-enriched regions, Ni signals are slightly attenuated, indicating that nano-sized L1_2_ ordered phases are in their early nucleation/growth stages without significant coarsening. This microstructure is consistent with the high UTS and notable elongation observed for the 700-1 condition. Cobalt is nearly uniformly distributed but shows slight enrichment near some grain boundaries, suggesting its role in tuning local stacking fault energy may be related to the activation of deformation twinning and cross-slip, explaining the higher initial work-hardening rate in the tensile curve.

**Figure 11:**
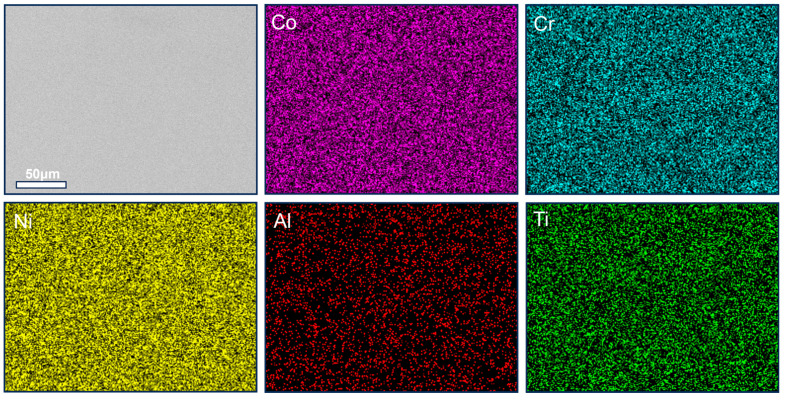
BSE image and corresponding EDS mapping of 700-1-550-24 MPEA.

Figure 11 displays EDS results for the alloy after the 700-1-550-24 treatment, revealing significant microstructural evolution compared to Figure 10. Dispersed bright precipitates appear near grain boundaries in the BSE image. Elemental maps confirm these are primarily enriched in Al and Ti, forming continuous, band-like structures along some grain boundaries. This indicates that the L1_2_ ordered phase has undergone significant coarsening and clustering during aging. These coarse precipitates enhance yield strength and UTS through dislocation cutting/bypass mechanisms but simultaneously deplete the plasticity reserve near grain boundaries, promoting the initiation and propagation of micro-cracks and leading to the reduction in TE from 10.2% to 9.4%. Furthermore, Co enrichment along grain boundaries is more pronounced in the 700-1-550-24 state, forming band-like or chain-like regions. This localized modification of stacking fault energy may further inhibit twinning and ε-martensite formation near boundaries, somewhat increasing the work-hardening capability, as suggested by the plateau in the tensile curve (Figure 9a). However, once the continuity and size of the Al–Ti-enriched bands exceed a critical threshold, brittle intergranular fracture begins to dominate. This microstructural nuance is a key reason for the model’s systematic overestimation of TE while accurately predicting UTS: macroscopic ductility is highly sensitive to local damage evolution at grain boundaries, which is challenging to capture fully with a feature set based on average volume fractions and parameters.

Despite the remaining uncertainty in TE prediction, the framework is still valuable as a screening tool: UTS is predicted with high reliability and can efficiently narrow down candidate compositions and processing routes. TE predictions should be interpreted primarily for relative ranking and trend identification rather than as absolute values, especially in the low-ductility regime where microstructural damage evolution is highly sensitive to features not included in the present descriptor set. Future improvements will incorporate microstructure-sensitive descriptors (e.g., grain size, precipitate fraction/morphology proxies) and/or a two-stage strategy that first classifies brittle vs. ductile responses before regression.

## 5. Conclusions

In this study, we established a curated database and an interpretable machine-learning workflow to link composition, thermodynamic stability and processing parameters with the room-temperature ultimate tensile strength (UTS) and total elongation (TE) of Fe-Co-Cr-Ni-Mn-Al-Ti MPEAs. By combining model selection, interpretability analysis, inverse design and targeted experiments, we quantitatively clarified key composition–processing–property couplings and extracted practical guidelines for achieving ultra-high strength with usable ductility. The main conclusions are summarized as follows:

(1) Among the six regression algorithms trained on the unified 18-dimensional feature space, XGBoost delivered the best overall accuracy and generalization for both UTS and TE, and was adopted for subsequent screening and inverse design. The prediction for UTS is robust across the studied strength range, whereas TE remains more difficult to capture—particularly in the low-ductility regime—highlighting the need for more microstructure-sensitive inputs.
(2) SHAP-based interpretation identifies Ti, Al and Cr as primary strength-promoting elements within the investigated processing window, consistent with the combined effects of solid-solution hardening, precipitation strengthening and lattice distortion. In addition, a moderate atomic-size mismatch (δ ~4%) is associated with a more favorable strength–ductility synergy, suggesting that carefully tuned size mismatch is beneficial while excessive mismatch can penalize plasticity.
(3) Guided by the trained model under physically constrained conditions, two candidate alloy/process routes were proposed and experimentally validated. The 700 °C/1 h annealed condition achieved a UTS of 1604 MPa with a TE of 10.2%, while additional aging at 550 °C for 24 h increased the UTS to 1694 MPa with a TE of 9.4%. Both conditions fall within the ultra-high-strength (≥1600 MPa) regime with near-10% ductility, supporting the reliability of the inverse-design strategy.
(4) The model–experiment comparison further suggests that a relatively high cooling rate (e.g., water or liquid-nitrogen quenching), annealing around 700 ± 20 °C and avoiding excessively long aging are effective levers to mitigate premature embrittlement while retaining high strength. The remaining deviations in TE prediction indicate that future improvements should incorporate explicit microstructural descriptors (e.g., grain size, precipitate fraction/morphology, interfacial energy and segregation-related metrics) to better quantify brittleness thresholds associated with phase transformations.

## Figures and Tables

**Table 1:** Feature generation equations. Table depicting the different formulae to calculate different features.

Feature Category	Parameter	Unit/Encoding	Physical Interpretation
Composition	Fe, Co, Cr, Ni, Mn	at.%	Solvent matrix effects; SFE modulation.
	Al, Ti	at.%	Solute strengthening. Precipitate formers (γ’-L1_2_).
Thermodynamics	VEC	-	Phase stability criterion (FCC vs. BCC/). Threshold ~8.0.
	$\delta =100\;\times \sqrt{\sum_{i=1}^{n}c_{i}{\left(1-\frac{r_{i}}{\overset{-}{r}}\right)}^{2}}$	%	Lattice distortion energy. Solution hardening potential.
	$\mathrm{Ave}.\mathrm{Elect}=\sum_{i=1}^{n}c_{i}\cdot \chi_{i}$	-	Average Electronegativity. The overall electron affinity of the alloy and the chemical bonding properties between atoms
	${\Delta S}_{\mathrm{mix}}=-R\sum_{i=1}^{n}c_{i}\ln c_{i}$	J/(mol·K)	Configurational Mixing Entropy. A higher mixing entropy can reduce the Gibbs free energy of the system (*G* = *H* − *TS*), thereby stabilizing the solid solution phase (such as FCC or BCC) at high temperatures and inhibiting the formation of intermetallic compounds.
Processing	Cooling Rate	K/s (Log scale)	Replaces ordinal codes. Controls vacancy retention & supersaturation. Values: Furnace (0.1), Air (5), Liquid Nitrogen (40), Water (500).
	Aging Temp/Time	°C/h	Kinetics of precipitation (Ostwald ripening control).
	Annealing temp/Time	°C/h	Recrystallization kinetics. Resuscitation–Recrystallization–Grain Growth and “Dissolution/Homogeneity”
	Homogenization Temp	°C/h	The degree of elimination of as-cast compositional segregation and the solubility tendency of coarse second phase.
	Rolling reduction	%	Cold working plastic deformation degree

**Table 2:** Using the optimal model XGBoost, two different MPEAs are designed through backpropagation.

Parameter	700-1	700-1-550-24
Fe(at%)	0	0
Co(at%)	30	30
Cr(at%)	15	15
Ni(at%)	45	45
Mn(at%)	0	0
Al(at%)	5	5
Ti(at%)	5	5
Homogenization temp. (°C)	1200	1200
Reduction rate (%)	80	80
Annealing temp. (°C)	700	700
Annealing time (h)	1	1
Cooling method	Water quenching (500)	Water quenching (500)
Aging temp. (°C)	0	550
Aging time (h)	0	24
Predicted UTS (MPa)	1711	1695
Predicted TE (%)	32	22

## Data Availability

The original contributions presented in this study are included in the article/Supplementary Materials. Further inquiries can be directed to the corresponding authors.

## Supplementary Materials

The following supporting information can be downloaded at: https://www.mdpi.com/article/10.3390/ma19020422/s1, Figure S1: Predictive chart of different holding times for HEA. It shows the predicted UTS and TE values for different holding times of 0, 1, 2, 4, 8, 12, and 24 h. The black line represents the predicted UTS value, and the red line represents the predicted TE value; Table S1: The databases collected in the text.
