# Supplementary material for: Design of Fe-Co-Cr-Ni-Mn-Al-Ti Multi-Principal Element Alloys Based on Machine Learning
Source: Materials (Basel). 2026 Jan 21;19(2):422. doi: 10.3390/ma19020422 (PMC12842681; doi:10.3390/ma19020422)
Supplement: Supplementary file 1 [file materials-19-00422-s001.zip › materials-4069462-supplementary.pdf]

Supplementary Materials

Design of Fe-Co-Cr-Ni-Mn-Al-Ti multi-principal element alloys based on machine learning

Xiaotian Xu [1], Zhongping He [1,*], Kaiyuan Zheng [1], Lun Che [1], Feng Zhao [2] and Deng Hua [3,*]

[1] School of Mechanical Engineering, Chengdu University, Chengdu 610106, China; xuxiaotian@stu.cdu.edu.cn (X.X.); zhengkaiyuan@stu.cdu.edu.cn (K.Z.); chelun@cdu.edu.cn (L.C.)

[2] Institute for Advanced Study, Chengdu University, Chengdu 610106, China; zhaofeng@cdu.edu.cn

[3] School of Artificial Intelligence and Electronic Engineering, Sichuan University of Business and Technology, Chengdu 611745, China

* Correspondence: hezhongping@cdu.edu.cn (Z.H.); huadeng1919@hotmail.com (D.H.)

1. The predicted conditions of alloys with the same composition under different tempering parameters

As shown in Fig S1, the model predicts that UST increases with the increase of aging time, while TE decreases rapidly with the increase of aging time. This proves that the model is sensitive to aging treatment.

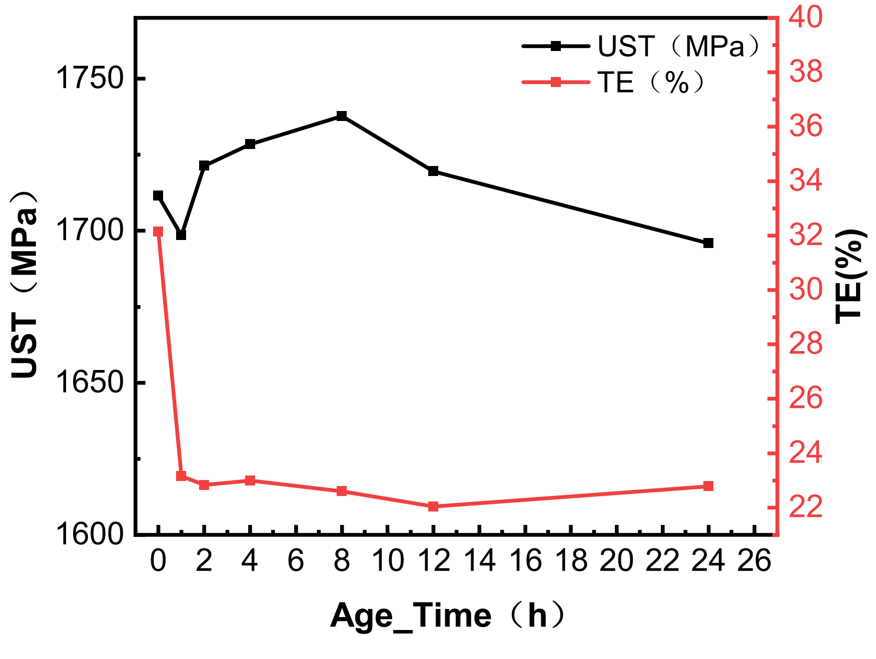

Fig S1. 700-1 Predictive chart of different holding times for HEA. It shows the predicted UTS and TE values for different holding times of 0, 1, 2, 4, 8, 12, and 24 hours. The black line represents the predicted UTS value, and the red line represents the predicted TE value.
